# Fusion of the ^1^H NMR data of serum, urine and exhaled breath condensate in order to discriminate chronic obstructive pulmonary disease and obstructive sleep apnea syndrome

**DOI:** 10.1007/s11306-015-0808-5

**Published:** 2015-05-22

**Authors:** Adam Ząbek, Ivana Stanimirova, Stanisław Deja, Wojciech Barg, Aneta Kowal, Anna Korzeniewska, Magdalena Orczyk-Pawiłowicz, Daniel Baranowski, Zofia Gdaniec, Renata Jankowska, Piotr Młynarz

**Affiliations:** 1 Department of Bioorganic Chemistry, Wroclaw University of Technology, 27 Wybrzeze Wyspianskiego Str., 50-370 Wroclaw, Poland; 2 Institute of Chemistry, University of Silesia, 9 Szkolna Str., 40-006 Katowice, Poland; 3 Faculty of Chemistry, Opole University, 11a Kopernik Sq., 45-040 Opole, Poland; 4 Department of Physiology, Wroclaw Medical University, 10 Chalubinskiego Str., 50-368 Wroclaw, Poland; 5 Department and Clinic of Pulmonology and Lung Cancers, Wroclaw Medical University, 105 Grabiszynska Str., 53-439 Wroclaw, Poland; 6 Department of Chemistry and Immunochemistry, Wroclaw Medical University, 44a Bujwida Str., 50-345, Wroclaw Poland; 7 Bioorganic Chemistry Institute, Polish Academy of Science, 12 Noskowskiego Str., 61-714 Poznan, Poland

**Keywords:** Chemometrics, Discriminant models, Chronic obstructive pulmonary disease (COPD), Obstructive sleep apnea syndrome (OSAS), NMR spectroscopy

## Abstract

Chronic obstructive pulmonary disease, COPD, affects the condition of the entire human organism and causes multiple comorbidities. Pathological lung changes lead to quantitative changes in the composition of the metabolites in different body fluids. The obstructive sleep apnea syndrome, OSAS, occurs in conjunction with chronic obstructive pulmonary disease in about 10–20 % of individuals who have COPD. Both conditions share the same comorbidities and this makes differentiating them difficult. The aim of this study was to investigate whether it is possible to diagnose a patient with either COPD or the OSA syndrome using a set of selected metabolites and to determine whether the metabolites that are present in one type of biofluid (serum, exhaled breath condensate or urine) or whether a combination of metabolites that are present in two biofluids or whether a set of metabolites that are present in all three biofluids are necessary to correctly diagnose a patient. A quantitative analysis of the metabolites in all three biofluid samples was performed using ^1^H NMR spectroscopy. A multivariate bootstrap approach that combines partial least squares regression with the variable importance in projection score (VIP-score) and selectivity ratio (SR) was adopted in order to construct discriminant diagnostic models for the groups of individuals with COPD and OSAS. A comparison study of all of the discriminant models that were constructed and validated showed that the discriminant partial least squares model using only ten urine metabolites (selected with the SR approach) has a specificity of 100 % and a sensitivity of 86.67 %. This model (AUC_test_ = 0.95) presented the best prediction performance. The main conclusion of this study is that urine metabolites, among the others, present the highest probability for correctly identifying patents with COPD and the lowest probability for an incorrect identification of the OSA syndrome as developed COPD. Another important conclusion is that the changes in the metabolite levels of exhaled breath condensates do not appear to be specific enough to differentiate between patients with COPD and OSAS.

**Electronic supplementary material:**

The online version of this article (doi:10.1007/s11306-015-0808-5) contains supplementary material, which is available to authorized users.

## Introduction

Chronic obstructive pulmonary disease, COPD, is a preventable and treatable disease that is characterized by a progressive and persistent airflow limitation which is the result of chronic inflammation (Global Strategy for Diagnosis, Management, and Prevention of COPD 2014). Pathological changes in COPD occur in small airways, lung parenchyma and small pulmonary vessels. Morphological changes in COPD include fibrosis and narrowing of small airways, together with parenchymal and alveolar destruction. This results in air trapping, emphysema, persistent lung hyperinflation and impaired exchange of gases (Hogg 2004; Baraldo et al. 2012). Consequently, patients with severe COPD suffer from respiratory insufficiency, pulmonary hypertension and right ventricular failure. A cornerstone of those morphological changes is an abnormal inflammatory response to noxious particles or gases with repeated tissue injury and repair (Górska et al. 2010; Barnes 2014). Inflammatory infiltrations are characterized by a cell pattern that has an increased number of alveolar macrophages, neutrophils and cytotoxic T-lymphocytes, which release various inflammatory mediators (Pappas et al. 2013; Barnes et al. 2003). The mechanism of amplifications and alterations in the inflammatory response in COPD patients probably depend on genetic and environmental factors that are not yet fully understood. An imbalance in proteases–antiproteases and repeated oxidative stress are involved in the process and biomarkers of oxidative stress are usually present in the biofluids (serum, exhaled breath condensate, sputum and urine) that are collected from COPD patients (Pillai et al. 2009; Castaldi et al. 2010; Kohansal et al. 2009; Stockley 2013; Vestbo and Rennard 2010).

Obstructive sleep apnea syndrome, OSAS, is defined as a sleep disorder in which an individual has 15 or more episodes of apnea or hypopnea per hour (apnea/hypopnea index, AHI ≥ 15) or AHI ≥ 5 with associated symptoms like fatigue, impaired cognition and/or increased daytime sleepiness (Park et al. 2011). The episodes of apnea typically last 20–40 s and result from an obstruction of the upper airways in adults, which is usually due to a pharyngeal collapse. Obesity is considered to be the most important predisposing factor as it causes an accumulation of fat in the peripharyngeal tissues (Romero-Corral et al. 2010; Tuomilehto et al. 2013). The OSA syndrome is often associated with other anatomical alterations that reduce the lumen of the pharynx, e.g. a thickening of the lateral parapharyngeal muscular walls or an increase in the length of the pharynx. The narrow airways are generally more prone to collapse than the larger ones (the Venturi effect) and this causes a further reduction of their lumen. The pharynx is kept patent mainly by the proper activity of dilator pharyngeal muscles. It was demonstrated that during sleep, this activity declines physiologically due to a decrease in the reflex mechanisms from chemoreceptors and mechanoreceptors. Consequently, while sleeping, the under stimulated muscles cannot always allow airflow in individuals with narrow upper airways, and the OSA syndrome occurs (Jordan and White 2008).

The pathogenic factors in both conditions are different and do not increase the risk of their incidence. The prevalence of COPD in the patients with the OSA syndrome is in the range of 10–20 %. However, COPD and OSAS share common comorbidities, especially cardiovascular diseases, which may be linked to the development of atherosclerosis. For this reason, there has been a growing interest in finding the chemical compounds (biomarkers) that reliably and unambiguously indicate COPD or OSAS in the recent years. A large number of the research works that are devoted to the high-throughput analysis of COPD have mainly been focused on a comparison of the metabolites in the exhaled breath condensate (EBC) of individuals with COPD and healthy controls (Bertini et al. 2014; Basanta et al. 2012; Fens et al. 2011; de Laurentiis et al. 2008). Only a few studies have described the results of such a comparison using plasma, serum and urine samples (Wang et al. 2013; McClay et al. 2010; Ubhi et al. 2012). Usually, special attention is paid to the smoking habits (smokers with or without emphysema) of the subjects who are being investigated (Paige et al. 2011; de Laurentiis et al. 2013; Ubhi et al. 2012). The collection of samples is often analyzed using ^1^H NMR, GC– and/or LC–MS. A metabolomic approach to the OSA syndrome involves a comparison of the LC–MS fingerprints that are obtained from plasma samples of patients who have been diagnosed with the sleep apnea or hypopnea syndrome, and healthy individuals (Ferrarini et al. 2013). Unsupervised methods like hierarchical clustering analysis, HCA, and principal component analysis, PCA, as well as supervised methods like discriminant partial least squares regression, PLS-DA, linear discriminant analysis, LDA, orthogonal partial least squares regression, OPLS-DA, and some recently proposed approaches such as the analysis of variance-principal component analysis, ANOVA-PCA and the analysis of variance-simultaneous component analysis have usually been adopted to describe the data structure or the discrimination of two or more groups of individuals. However, the selection of important biomarkers or the signal intervals that are important for the distinction between disease entities is often done using a univariate approach like the *t* test, the Fisher test or ANOVA. Subsequently, the set of important variables that has been selected is used to build a multivariate discriminant/classification model. Such a univariate approach does not allow for the selection of a set of potential biomarkers that are characteristic for the discrimination, because the variable selection is not performed during the construction of the discriminant or classification model. In our work, we offer a more comprehensive approach that uses the principles of metabolomic data fusion (Bro et al. 2013) and multivariate variable selection in order to build diagnostic models for patients with the OSA syndrome and/or COPD. The variables (metabolites that are analyzed in serum, exhaled breath condensate and urine) that are relevant to the two-group discrimination were identified using the bootstrap PLS-DA procedure combined with the variable importance in projection score, VIP-score, (Andersen and Bro 2010; Gosselin et al. 2010) or the selectivity ratio (SR) (Kvalheim and Karstang 1989; Rajalahti et al. 2009). The SR approach in PLS-DA has gained much popularity in recent years (Kvalheim et al. 2014; Kvalheim 2010), because the possibility of selecting variables that are large in absolute size, but that are not related to the discrimination of the model groups, is eliminated throughout the so-called target projection or target rotation transformation. With the target projection transformation, several PLS-DA components (the model’s complexity) are represented by a single predictive component that is unrelated to the orthogonal variation with the response variable. The same objective is met by the OPLS method, even though it uses a different algorithmic procedure. The interest in the SR method can also be explained by the fact that the predictive component for OPLS and PLS post-processing by similarity transformation (Ergon 2005) is identical to the predictive component that is obtained from the target projection transformation except for the scaling factor (Kvalheim et al. 2009). On the other hand, the variables that are selected using the VIP-score are related to both the response variable and to the variance of independent variables.

The bootstrap PLS-DA methodology combined with an estimation of VIP-scores and SRs for different sets of metabolites was proposed here to investigate: (i) whether it is possible to diagnose a patient with either the COPD disease or the OSA syndrome using a set of selected metabolites and to determine what a probability of false diagnostic decision is; (ii) whether the metabolites that are present in one type of biofluid (serum, exhaled breath condensate or urine) are sufficient enough for this diagnosis; (iii) whether a combination of metabolites that are present in two biofluids or a set of metabolites that is present in all three biofluids are necessary to correctly diagnose a patient (at a certain level of significance).

## Materials and methods

### Ethics statement

The study was conducted in agreement with the Declaration of Helsinki and was approved by the Ethics Committee of the Medical University in Wroclaw, Poland. All of the participants signed an informed consent form (KB-12/2010).

### Study population comprises

A total of 85 serum, 91 urine and 82 exhaled breath condensate samples were collected from adult individuals who had been diagnosed according to the generally accepted criteria. Over half of the individuals who were studied have concomitant cardiovascular disease (CVD) including ischemic heart disease and/or arterial hypertension and/or have suffered a brain stroke. All of these comorbidities were controlled during the study. Patients with any other unstable or acute diseases were excluded from the study. Finally, 46 individuals (18 patients with COPD and 28 patients with the OSA syndrome) who had had all three biofluids collected were included in the following targeted metabolomic data fusion analysis. The demographic data of those patients are presented in Table 1.

**Table 1:** Demographic data and clinical profiles of patients included in the study

	COPD	OSA
Number of patients	18	28
Sex (male/female)	9/9	23/5
Age (mean/range)	64/(49–81)	54/(27–65)
Body mass index (mean/range)	30/(20–33)	25/(22–41)

### Preparation of the samples for proton NMR spectroscopy

Samples of serum, urine and EBC were collected from the subjects participating in the study in the morning after they had fasted for at least eight hours. Serum was sampled from the peripheral vein and centrifuged for 10 min at 4000×*g*. EBC was collected using the EcoScreen Turbo (VIASYS Healthcare GmbH, Hoechberg, Germany) apparatus according to the manufacturer’s instructions. The subjects were without a previous oral hygiene and breathed spontaneously through a mouthpiece while sitting upright and wearing a nose clip. The sampling procedure was finished when the EBC sample volume was at least 2 mL. All of the samples were frozen in liquid nitrogen immediately after collection and stored at −80 °C until the analysis.

Prior to the metabolomic experiment, the serum samples were thawed at room temperature and vortexed. Next, mixtures of 200 μL of serum and 400 μL of saline solution (prepared from 0.9 % NaCl, 15 % D_2_O and 3 mM TSP) were mixed again. After centrifugation (12,000×*g*, 10 min), an aliquot of 550 μL of each sample supernatant was subsequently transferred into a 5 mm NMR tubes. Samples were kept at 4 °C until the measurement.

All urine samples were thawed at room temperature and mixed using a vortex mixer. The samples were centrifuged for 10 min at 12,000×g and 400 μL of supernatant was then transferred into a new Eppendorf tube. Next, the samples were mixed with 200 μL of PBS (0.5 M, pH 7.00, 33 % D_2_O, 3 mM NaN_3_ and 3 mM TSP). The samples were mixed again and finally, an aliquot of 550 μL was transferred into a 5 mm NMR tube.

The EBC samples were thawed at room temperature and mixed using a vortex mixer. Aliquots of 250 μL D_2_O (3 mM TSP, 3 mM NaN_3_) were added to 300 μL EBC. After centrifugation (10,000×*g* for 10 min), 500 μL samples of the clarified solutions were transferred into 5 mm NMR tubes.

### ^1^H NMR measurements

The NMR spectra of the serum and urine samples were recorded at 300 K using an Avance II spectrometer (Bruker, GmBH, Germany) operating at a proton frequency of 600.58 MHz, while the NMR spectra of the EBC samples were recorded at 300 K using an Avance III spectrometer (Bruker, GmBH, Germany) operating at proton QCI CryoProbe frequency of 700 MHz.

The NMR spectra of the serums were recorded by using a CPMG pulse sequence with water presaturation on a Bruker notation. For each sample, 128 sequential scans were collected with spin-echo delay of 400 μs; 80 loops; a relaxation delay of 3.5 s; an acquisition time of 2.73 s; TD of 64 k; SW of 20.01 ppm.

The NMR spectra of the urine samples were recorded using nuclear Overhauser effect spectroscopy, NOESY pulse sequence with water presaturation on a Bruker notation: a relaxation delay of 3.5 s; an acquisition time of 1.36 s; 128 transients; TD of 32 k; SW of 20.01 ppm.

The NMR spectra of the EBC samples were recorded using the excitation sculpting (ZGESGP) pulse sequence with water presaturation on a Bruker notation: a relaxation delay of 3.5 s; an acquisition time of 2.32 s; 256 transients; TD of 64 k; SW of 20.01 ppm. This excitation sculpting (ZGESGP) pulse sequence allowed obtaining the best water signal quenching and recording the high quality ^1^H NMR spectra. Spectra were processed with line broadening of 0.3 Hz and manually phased and baseline corrected using Topspin 1.3 software (Bruker, GmBH, Germany) and referenced to α-glucose signal *δ* = 5.225 ppm for the serum samples and to the TSP resonance at *δ* = 0.0 ppm for the urine and EBC samples. The correction of peak positions (alignment) was done using the correlation optimized warping algorithm, COW, and the *i*coshift algorithm implemented in Matlab (Matlab v. 8.1, Mathworks Inc.). The spectra were normalized using the Probabilistic Quotient Normalization (PQN) method. Finally, the dataset was binned into 14,375 integrals (serum) of an equal width (0.001 ppm), 14,625 integrals (urine) of equal width (0.005 ppm) and 14,125 integrals (EBC) of an equal width (0.001 ppm).

### Preprocessing of variables prior to analysis

**Fig. 1:**
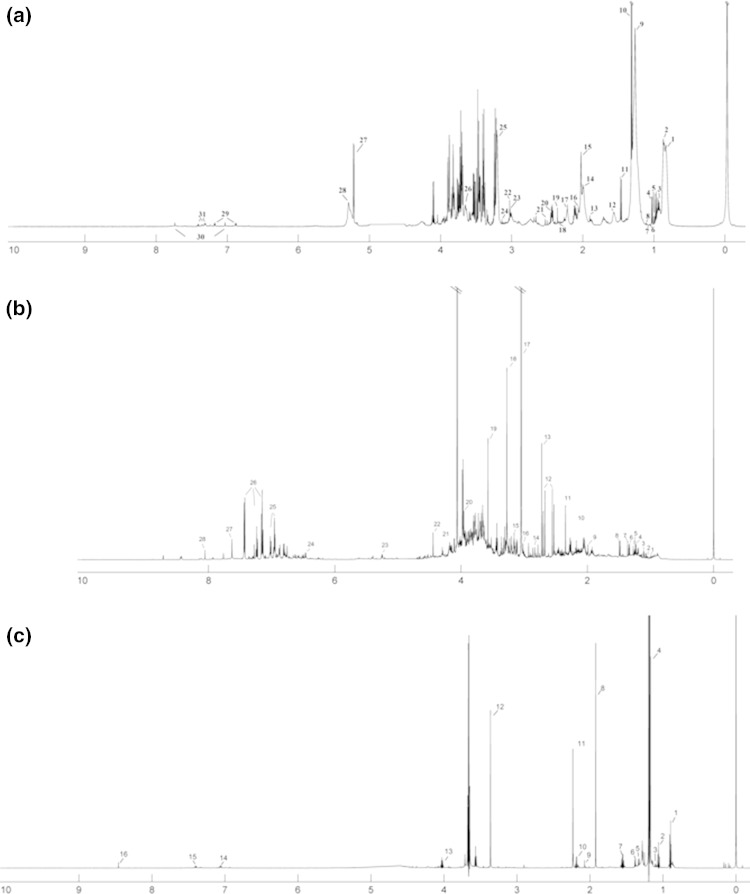
The median of ^1^H NMR spectra of the** a** serum COPD samples: *1a* L1; *2a* L2; *3a* Leucine; *4a* Valine; *5a* Isoleucine; *6a* Isobutyrate; *7a* Unk_1; *8a* 3-Hydroxybutyrate; *9a* L3; *10a* Lactate; *11a* Alanine; *12a* L4; *13a* Acetate; *14a* L5; *15a* NAC1; *16a* NAC2; *17a* Unk2; *18a* Pyruvate; *19a* Succinate; *20a* Glutamine; *21a* Citrate; *22a* Creatine; *23a* Creatinine; *24a* Choline; *25a* GPC + APC; *26a* Unk_2; *27a* Glucose; *28a* L6; *29a* Tyrosine; *30a* Histidine; *31a* Phenylalanine; **b** urine COPD samples: *1b* Isobutyrate; *2b* Methylsuccinate; *3b* 3-Aminoisobutyrate; *4b* Methylmalonate; *5b* 3-Hydroxyisovalerate; *6b* Lactate; *7b* 2-Hydroxyisobutyrate; *8b* Alanine; *9b* Acetate; *10b* Unk_1; *11b* Unk_2; *12b* Citrate; *13b* Dimethylamine; *14b* *N*,*N*-Dimethylformamide; *15b* sn-Glycero-3-phosphocholine; *16b* Creatine; *17b* Creatinine; *18b* Trimethylamine *N*-oxide; *19b* Glycine; *20b* Glycolate; *21b* Unk_3; *22b* Trigonelline; *23b* cis_Aconitate; *24b* Hydroxyphenyl; *25b* *N*-Phenylacetylglycine; *26b* Hippurate; *27b* Xanthine; *28b* Formate; **c** EBC COPD samples: *1c* Butyrate; *2c* Propionate; *3c* Propylene glycol; *4c* Ethanol; *5c* 3-Hydroxyisovalerate; *6c* acetate; *7c* Unk_1; *8c* Acetate; *9c* Acetone; *10c* Unk_2; *11c* Methanol; *12c* Unk_3; *13c* Isopropanol; *14c* Phenol; *15c* Unk_4; *16c* Formate

A total of 31 serum, 27 urine and 16 EBC metabolites were analyzed. The concentration of any metabolite was obtained using NMR as a signal integral of the non-overlapping resonances (or a cluster of partly overlapping resonances). The metabolite resonances were identified according to assignments published in the literature and in on-line databases (Biological Magnetic Resonance Data Bank and Human Metabolome Data Base). The median ^1^H NMR spectra of serum, urine and EBC in individuals with COPD are presented in Fig. 1.

### Discriminant analysis for the identification of biomarkers

**Fig. 2:**
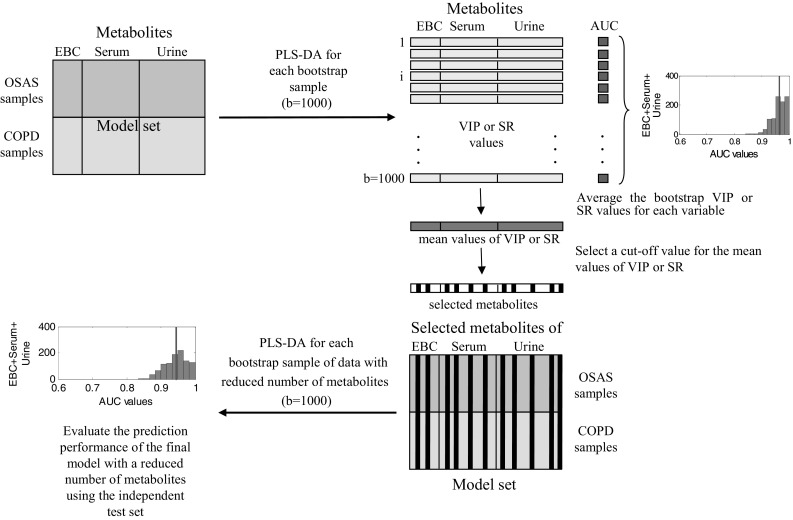
A general scheme of the data analysis procedure with the main steps highlighted. The methodology is illustrated on a data set containing the metabolites of EBC, serum and urine biofluids

The discriminant version of the Partial Least Squares regression with the bootstrap procedure for estimating the quality of the models with selected variables was adopted and the prediction for a test set was estimated. The model samples were chosen with the Kennard and Stone algorithm applied separately to each group in order to guarantee the representativity of the model set and to avoid the possibility of having outlying samples in the test set. The autoscaled (variables of all three biofluid blocks) data set for each group was considered in the Kennard and Stone algorithm, since the Euclidean distance is used as a similarity measure between two samples. The model set should also be balanced (containing the same number of samples from each group) in order to avoid the weighting of the discriminant cut-off value for the response variable (Brereton and Lloyd 2014). Therefore, 13 samples (75 % of the samples from the less numerous group) were selected from each group. The remaining samples (15 OSAS samples and five COPD samples) formed the test set. As was mentioned earlier, in order to reduce the chances of overfitting due to the larger number of variables with respect to the number of samples mainly in the two- and three-block PLS-DA models and to enable the easier interpretation of the models, variable selection using the VIP-scores (Andersen and Bro 2010; Gosselin et al. 2010; Kvalheim and Karstang 1989) or SR (Rajalahti et al. 2009) was performed. The VIP-score is a quantitative measure that indicates the contribution of a single variable to the description of both independent variables and the response variable, while the SR is ratio of the explained variance to the residual variance of a variable after target projection transformation. The VIP-score and SR for each variable were estimated 1000 times using the bootstrap procedure with a replacement. The two procedures will be abbreviated as VIP-PLS-DA or SR-PLS-D in the rest of the text. The main steps of the data modeling procedure are presented in Fig. 2. This general methodology was also followed in the analysis of data containing the metabolites that are present in one or two biofluids.

The variables that had an average VIP-scores or SRs below a given cut-off value were discarded from the final model. The selection of an appropriate cut-off value for VIPs or SRs is an important issue. In general, a variable that has a unitary VIP-score is highly influential since the average of the squared values of the VIPs is equal to 1.0. Even though the unitary cut-off value is often used, some researchers have found it to be too restrictive. Other authors have stressed that this value depends on the data structure and that an important variable may have a VIP-score of more than 0.8. Here, we have chosen a cut-off value of 0.8 after a preliminary investigation of the uncertainty in the estimation of the VIPs. A similar bootstrap VIP-PLS-DA methodology was also used for the selection of wavelengths in a spectral imaging dataset (Kvalheim and Karstang 1989). Moreover, some authors (Andersen and Bro 2010) have pointed out that applying a variable selection that is based on the VIP-scores only once is usually ineffective due to the large number of variables that remain and therefore, it has been proposed that the selection procedure be repeated several times. In this research work, we repeated the whole VIP procedure three times. Thus, for each bootstrap sample (a sample formed by re-sampling the original data populations with a replacement) of the model set, a PLS-DA model of certain complexity that was chosen based on a leave-one-out cross-validation procedure is selected and the VIP-scores or SR after the target projection transformation (Rajalahti et al. 2009) were calculated. After considering 1000 bootstrap samples, the average value of the area under the receiver operating curve (AUC) was calculated as a figure of merit that described the model’s performance. The standard error (uncertainty) in the AUC estimation (se)_b_, with b bootstrap samples (b = 1 000) is defined as follows:

1 $$ ( {\text{se)}}_{\text{b}} = \sqrt {\frac{{\sum\limits_{{{\text{i}} = 1}}^{\text{b}} { (\theta_{\text{i}}^{ *} - \bar{\theta }^{ *} )^{ 2} } }}{{{\text{b}} - 1}}} $$

In this equation, $\theta_{\text{i}}^{ *}$ is the estimate of the AUC for the i-th bootstrap sample and $\bar{\theta }^{ *}$ is the mean estimate of the AUC for all of the bootstrap samples.

The variables with average VIP-scores below 0.8 were removed. The cut-off value for SR can be estimated using the F-test, since SR is defined as the ratio between the variable variance that is explained in the PLS-DA model of a certain complexity and its residual variance after target projection transformation. Since the values of the F-distribution tend to 1.0, a unitary cut-off value can be used. In this work, we selected the cut-off value of SR based on the so-called discriminating variable test, the DIVA test, and the SR plots that have been proposed in the literature (Kvalheim et al. 2014). Unlike the VIP-PLS-DA, the bootstrap SR-PLS-DA methodology was applied once to each dataset and variables with an average SRs found below a given cut-of value, which were chosen after an inspection of the DIVA and SR plots, were discarded from the final model. The prediction performance of the final model was estimated using the independent test set, which was not used during the construction of the model and variable selection. The respective AUC value, sensitivity, specificity and efficiency for the test set were also calculated. For the two-group problem that was studied in this work, sensitivity is defined as the percentage of samples from the OSAS group of patients that were correctly predicted by the model, while specificity is the percentage of samples that were collected from patents with COPD that were properly predicted as having COPD. The best model would have a sensitivity and a specificity of 100 %. One can also define the so-called efficiency, also known in the literature as the non-error rate, which is the total percentage of test samples that are correctly classified.

All calculations using in-house implemented routines were performed with MATLAB 7.0 (R14) on a personal computer (Intel(R) Pentium(R) M, 1.60 GHz with 2 GB RAM) using the Microsoft Windows XP (service pack 2) operating system.

## Results and discussion

Several discriminant models were built. Firstly the quality of the models for the individual blocks of variables (EBC, serum, urine), two blocks of variables and the three-block variables were evaluated using the bootstrap procedure with a replacement. The histograms of the AUC values that were obtained from the bootstrap procedure (the average AUC value for each model is shown as a vertical red line) are presented in Fig. 1S (Supplementary materials) and Table 2, while the sensitivity, specificity and efficiency of prediction are listed in Table 3.

**Table 2:** The average AUC values (±uncertainty in the AUC estimation) for the model set and the AUC values for the test set obtained from PLS-DA with all variables

Variables	Average AUC values for model set	AUC_test_
EBC	0.92 ± 0.05	0.52
Serum	0.88 ± 0.06	0.91
Urine	0.94 ± 0.04	0.93
EBC + serum	0.94 ± 0.04	0.91
EBC + urine	0.98 ± 0.02	0.81
Serum + urine	0.94 ± 0.04	0.95
EBC + serum + urine	0.96 ± 0.03	0.91

**Table 3:** Sensitivity, specificity and efficiency for the test set of the PLS-DA model with all variables

Variables	PLS-DA (complexity)	Sensitivity (%)	Specificity (%)	Efficiency (%)
EBC	1	80.00	20.00	65.00
Serum	1	73.33	100.00	80.00
Urine	1	86.67	100.00	90.00
EBC + serum	1	73.33	80.00	75.00
EBC + urine	1	80.00	60.00	75.00
Serum + urine	1	73.33	100.00	80.00
EBC + serum + u rine	1	66.67	80.00	70.00

From the values that are presented in Tables 2 and 3, one can conclude that the models that solely exploit the serum or urine variables show relatively good prediction capabilities (AUC_test_(serum) = 0.91 and AUC_test_(urine) = 0.93). Four OSAS samples were incorrectly predicted as COPD samples using serum variables, which results in a sensitivity of 73.33 %, while only two OSAS samples (a sensitivity of 86.67 %) were wrongly predicted by the model using all of the urine variables. Both models show the highest specificity of 100 % thus indicating the best prediction of the COPD samples. The uncertainty in the AUC estimation of the serum model is larger than the uncertainty that was obtained for the urine model (Fig. 1S; Table 2). The model using only EBC variables had a poor prediction performance (AUC_test_ = 0.52), which indicates that there are some differences between the model and test samples. In fact, the model has a relatively high sensitivity of 80 %, but a very low specificity of 20 %. This suggests that the probability of the correct identification of a patient with the OSA syndrome is high with this EBC model, although the probability of a correct COPD identification is very low. Thus, there is a high risk that a patient with developed COPD may be diagnosed with the OSA syndrome using this model. The models that combine the EBC variables with either serum or urine metabolites have somewhat lower specificities in comparison to the models that were built using all of the serum or urine metabolites only. Compared to the model that used only the serum metabolites, the model using both EBC and serum metabolites had the same sensitivity and a lower specificity of 80 %. This suggests that the probability of identifying a COPD patient as a patient with the OSA syndrome is higher with the model of the two types of metabolites than the probability that is estimated with the model using only the serum metabolites. The model using both EBC and urine metabolites presents a slightly lower sensitivity of 80 % and a poorer specificity of 60 % in comparison to the model that was built for the urine metabolites only. This indicates that the inclusion of the EBC variables results in an incorrect prediction of the COPD samples as the OSA samples. The model using both the serum and urine metabolites, which had a sensitivity of 73.33 % and a specificity of 100 %, had a comparable prediction performance (AUC_test_ = 0.95) to the models that were built for either the serum or urine metabolites. However, from a practical point of view, the analysis of one biofluid is the easiest and the most preferable. The main question is whether a limited number of variables (possible biomarkers) would still provide a good discrimination of the two groups of patients that were studied and a good prediction performance of the models. The average AUC values for the model sets and the respective test sets with different sets of metabolites, which were obtained using the VIP-PLS-DA and SR-PLS-DA methods, are presented in Table 4. The cut-off values for the average SRs are also presented therein. As was mentioned earlier, the cut-off values for the average SRs were determined using the so-called discriminating variable test, the DIVA test. The DIVA test is a nonparametric test in which the relation of the mean correct classification rate, MCCR, for variables found in a given SR interval is examined. The mean correct classification rate increases with the increasing values of SR which provides a quantitative measure of the discriminatory ability in the whole range of SR intervals (Rajalahti et al. 2009). The values of the prediction figures of merit for the sets of metabolites are shown in Table 5 and the respective histograms for several selected models are shown in Fig. 2S (Supplementary materials).

**Table 4:** The AUC values for the model (±uncertainty in the AUC estimation) and test sets with selected variables from VIP-PLS-DA and SR-PLS-DA

Variables	Variable selection using VIP-PLS-DA		Variable selection using SR-PLS-DA		
	Average AUC values for model set	AUC_test_	Average AUC values for model set	AUC_test_	Cut-off value of SR (MCCR [%])
EBC	0.93 ± 0.05	0.48	0.90 ± 0.06	0.48	0.3 (60)
Serum	0.87 ± 0.06	0.92	0.97 ± 0.03	0.88	0.8 (62)
Urine	0.98 ± 0.02	0.83	0.90 ± 0.06	0.95	0.4 (60)
EBC + serum	0.92 ± 0.04	0.85	0.97 ± 0.03	0.88	0.8 (62)
EBC + urine	0.99 ± 0.01	0.63	0.91 ± 0.05	0.89	0.4 (60)
Serum + urine	0.92 ± 0.05	0.93	0.88 ± 0.05	0.93	0.5 (61)
EBC + serum + urine	0.94 ± 0.03	0.92	0.97 ± 0.03	0.91	0.6 (62)

The mean correct classification rates, MCCRs, which were estimated for the cut-off values of the average SRs, are also listed

**Table 5:** Sensitivity, specificity and efficiency for the test sets with variables selected by VIP-PLS-DA and SR-PLS-DA

Variables	VIP-PLS-DA (complexity)	SR-PLS-DA (complexity)	Sensitivity (%)		Specificity (%)		Efficiency (%)	
			VIP-PLS-DA	SR-PLS-DA	VIP-PLS-DA	SR-PLS-DA	VIP-PLS-DA	SR-PLS-DA
EBC	1	1	80.00	73.33	20.00	20.00	65.00	60.00
Serum	1	2	73.33	86.67	80.00	80.00	75.00	85.00
Urine	2	1	73.33	86.67	80.00	100.0	75.00	90.00
EBC + serum	1	2	73.33	86.67	80.00	80.00	75.00	85.00
EBC + urine	1	1	66.67	80.00	60.00	60.00	65.00	75.00
Serum + urine	1	1	66.67	73.33	80.00	100.0	70.00	80.00
EBC + serum + urine	1	2	60.00	86.67	80.00	60.00	65.00	80.00

The optimal complexities of the final models are also listed

Reducing the number of EBC metabolites based on the average VIP-scores and SRs that were obtained from the bootstrap PLS-DA method did not result in a better identification of individuals with COPD, which was indicated by the poor specificities of 20 % (Table 5). The same predictive performance, a sensitivity of 73.33 % and a specificity of 80.00 %, was observed for the models that were constructed with either the serum or urine variables that were found using VIP-PLS-DA. Compared to VIP-PLS-DA, the PLS-DA model using a subset of urine metabolites that was obtained using the SR procedure, had a slightly improved sensitivity and specificity of 86.67 and 100 %, respectively, while the model that was built for a subset of serum metabolites had only a slightly improved sensitivity. Serum and urine body fluids contain different metabolites, but both of the subsets that were obtained using VIP-PLS-DA showed the same potential to distinguish between individuals with COPD and those that had been diagnosed with the OSA syndrome. The subsets of serum metabolites that were found using the SR and VIP methods contained the same eleven variables (see Table 6), although it appears that the inclusion of L2, Leucine, Lactate, L6, NAC1, NAC2 and the removal of L1 and GPC + APC serum metabolites leads to an improvement in the model’s prediction.

**Table 6:** Variables selected by the VIP-PLS-DA and SR-PLS-DA methods in all models constructed

Block(s) of variables	Variables selected using VIP-PLS-DA	Variables selected using SR-PLS-DA	Percentage of common variables
EBC	Propylene glycol, ethanol, 3-hydroxyisovalerate, acetone, methanol, Unk2 (δ = 2.90 ppm)^a^, Unk3 (δ = 3.57 ppm), Unk4 (δ = 7.07 ppm), formate	Propylene glycol, ethanol, 3-hydroxyisovalerate, methanol, Unk2 (δ = 2.90 ppm), Unk3 (δ = 3.57 ppm), isopropanol, formate	44 (7 vars)
Serum	L1, L3, L4, L6, isoleucine, Unk1 (δ = 1.11 ppm), Unk2 (δ = 2.22 ppm), Unk3 (δ = 4.26 ppm), acetate, glutamine, choline, GPC + APC, histidine, phenylalanine	L2, L3, L4, L6, leucine, isoleucine, Unk1 (δ = 1.11 ppm), Unk2 (δ = 2.22 ppm), Unk3 (δ = 4.26 ppm), lactate, acetate, L6, NAC1, NAC2, glutamine, choline, histidine, phenylalanine	39 (12 vars)
Urine	Isobutyrate, 3-aminoisobutyrate, 2-hydroxyisobutyrate, Unk2 (δ = 2.35 ppm), *N,N*-dimethylglycine, sn-glycero-3-phosphocholine, creatine, creatinine, xanthine, Formate	Isobutyrate, methylsuccinate, 3-hydroxyisovalerate, lactate, 2-hydroxyisobutyrate, Unk2 (δ = 2.35 ppm), *N,N*-dimethylglycine, sn-glycero-3-phosphocholine, cis_Aconitate, Formate	18 (5 vars)
EBC+	Propylene glycol, 3-Hydroxyisovalerate, Methanol, Formate +		23 (11 vars)
Serum	L1, L3, L4, L6, isoleucine, Unk1 (δ = 1.11 ppm), Unk3 (δ = 4.26 ppm), acetate, choline, glutamine, GPC + APC, histidine, phenylalanine	L2, L3, L4, L6, leucine, isoleucine, Unk1 (δ = 1.11 ppm), Unk2 (δ = 2.22 ppm), Unk3 (δ = 4.26 ppm), lactate, acetate, L6, NAC1, NAC2, glutamine, choline, histidine, phenylalanine	
EBC+	propylene glycol, ethanol, 3-Hydroxyisovalerate, Unk2 (δ = 2.90 ppm), methanol, isopropanol, formate +	Propylene glycol, formate +	18 (8 vars)
Urine	Isobutyrate, 3-aminoisobutyrate, 2-hydroxyisobutyrate, Unk2 (δ = 2.35 ppm), *N,N*-dimethylglycine, sn-glycero-3-phosphocholine, creatine, creatinine, trimethylamine *N*-oxide, xanthine, formate	Isobutyrate, methylsuccinate, methylmalonate, 3-hydroxyisovalerate, lactate, 2-hydroxyisobutyrate, Unk2 (δ = 2.35 ppm), *N,N*-dimethylglycine, sn-glycero-3-phosphocholine, cis_aconitate, formate	
Serum+	L1, L3, L4, L6, isoleucine, Unk1 (δ = 1.11 ppm), Unk3 (δ = 4.26 ppm), acetate, choline, glutamine, GPC + APC, histidine, phenylalanine	L2, L3, L4, L6, leucine, isoleucine, Unk1 (δ = 1.11 ppm), Unk2 (δ = 2.22 ppm), Unk3 (δ = 4.26 ppm), isobutyrate, lactate, acetate, L6, NAC1, NAC2, glutamine, citrate, creatinine, choline, GPC + APC, histidine, phenylalanine +	25 (15 vars)
Urine	Isobutyrate, 2-hydroxyisobutyrate, *N,N*-dimethylglycine, sn-glycero-3-phosphocholine, creatine, creatinine, formate	2-Hydroxyisobutyrate, Unk2 (δ = 2.35 ppm), *N,N*-dimethylglycine, sn-glycero-3-phosphocholine	
EBC+	Propylene glycol, 3-Hydroxyisovalerate, Methanol, Formate +		20 (15 vars)
Serum+	L1, L2, L3, L4, L6, valine, isoleucine, Unk1 (δ = 1.11 ppm), Unk_2 (δ = 2.22 ppm), Unk3 (δ = 4.26 ppm), acetate, glutamine, choline, GPC + APC, histidine, phenylalanine +	L1, L2, L3, L4, L6, isoleucine, Unk1 (δ = 1.11 ppm), Unk2 (δ = 2.22 ppm), Unk3 (δ = 4.26 ppm), lactate, acetate, glutamine, choline, L6, NAC_1,NAC_2, citrate, GPC + APC, histidine, phenylalanine +	
Urine	Isobutyrate, 2-hydroxyisobutyrate, *N,N*-dimethylglycine, sn-glycero-3-phosphocholine, creatine, creatinine, formate	*N,N*-Dimethylglycine	

^a^The notation Unk2 (δ = 2.90 ppm) means an unknown metabolite at a chemical shift of 2.90 ppm

Moreover the larger number of urine metabolites that were selected using the SR approach in comparison to VIP-PLS-DA as well as the fact that only five variables were found to be common for both sets of urine metabolites may explain the improved value of specificity.

Several important observations are apparent when comparing the prediction abilities of models with all of the variables and the reduced number of two-block variables. Compared to the model with all EBC and serum metabolites, the model with the subset of EBC and serum metabolites that were found using SR-PLS-DA had an improved sensitivity of 86.67 % and the same specificity of 80.00 %. The model using a subset of serum variables that were selected using the SR method had the same performance. In fact, none of the EBC metabolites were selected in the PLS-DA model and the serum metabolites were the same as those found using the SR-PLS-DA that was built for serum metabolites only. This confirms the previous observation that the EBC metabolites have a lower potential for the correct discrimination of COPD and OSAS patients than the serum metabolites.

The model with the EBC and urine variables that were selected with the SRs over 0.4 (see Tables 3, 5) had the same prediction performance as the model using all of the EBC and urine metabolites. Only two EBC metabolites (Propylene glycol, Formate +) were considered in this model (see Table 6). These two EBC metabolites appear to be strongly related to the development of the OSA syndrome in patients. In contrast, the model with the EBC and urine metabolites that had the largest VIP scores had a poor prediction performance (AUC_test_ = 0.63) with a low sensitivity and specificity of 66.67 and 60.00 %, respectively.

The model that was built for serum and urine metabolites that were selected using SR-PLS-DA had the same prediction features (sensitivity of 73.33 % and a specificity of 100 %) as the one that was constructed for all of the serum and urine metabolites. Compared to these models, the model using only ten urine metabolites that were selected from SR-PLS-DA also showed a specificity of 100 % although it had a better sensitivity of 86.67 %. Specifically, this model (AUC_test_ = 0.95) had the best prediction performance in comparison to all of the other models that were constructed (Tables 4, 5).

In general, it appears that urine metabolites present the highest probability for the correct identification of individuals with COPD and the lowest probability for the incorrect identification of the OSA syndrome as developed COPD. Specifically, the results showed that only ten urine metabolites may be sufficient for the development of a metabolomic diagnostic procedure. It should be pointed out that the collection of samples was not large enough to draw general conclusions and a larger set of samples will be necessary for the further validation of this procedure. Moreover, several studies have emphasized the possibility of using changes in the EBC metabolite levels for the correct identification of individuals with OSAS or individuals with COPD from healthy individuals. The results of this study indicate that changes in the level of EBC metabolites may not be specific enough to correctly identify COPD patients from individuals with OSAS and therefore, a large number of false positive identifications may occur.

## Concluding remarks

The main conclusion of this study is that only ten urine metabolites are enough to distinguish COPD patients from those with the OSA syndrome. The urine metabolites were selected using the SR approach. The model with a specificity of 100 % and a sensitivity of 86.67 % also presents the best prediction performance (AUC_test_ = 0.95) in comparison to all of the other models that were constructed. It appears that a combination of two biofluid metabolites or metabolites of all three types of biofluids is unnecessary to obtain a diagnostic model with improved predictive abilities. Perhaps a surprising conclusion is that changes in the concentration in the EBC metabolites were not specific enough to predict correctly the COPD or OSAS in individuals, which was illustrated by the poor performance of the discriminant models that were constructed for those variables.

## Electronic supplementary material

Below is the link to the electronic supplementary material.

Fig. 1S Histograms of the AUC values that were obtained from the bootstrapped PLS-DA procedure using a) all EBC variables, b) all serum variables and c) all urine variables, d) EBC and serum variables, e) EBC and urine variables f) serum and urine variables and g) all three-block variables. The average AUC value is represented by the vertical red line. Supplementary material 1 (TIFF 176 kb)

Fig. 2S Histograms of the AUC values that were obtained from the models with selected variables using a) the VIP-PLS-DA method of serum variables, b) the VIP-PLS-DA method of serum and urine variables, c) the SR-PLS-DA of urine variables and d) the SR-PLS-DA of serum and urine variables. Supplementary material 2 (TIFF 116 kb)

## Abbreviations

COPD: Chronic obstructive pulmonary disease
HCA: Hierarchical clustering analysis
OSAS: Obstructive sleep apnea syndrome
NMR: Nuclear magnetic resonance
EBC: Exhaled breath condensate
GC/LC–MS: Gas/liquid chromatography–mass spectrometry
PCA: Principal component analysis
PLS-DA: Partial least squares-discriminant analysis
LDA: Linear discriminant analysis
OPLS: Orthogonal partial least squares
OPLS-DA: Orthogonal partial least squares-discriminant analysis
ANOVA-PCA: Analysis of variance-principal component analysis
ANOVA-SCA: Analysis of variance-simultaneous component analysis
VIP-score: Variable importance in projection-score
SR: Selectivity ratio
TSP: 3-(Trimethylsilyl)-2,2′,3,3′-tetradeuteropropionate sodium salt TSP-d4
AUC: Area under the curve
DIVA test: Discriminating variable test
L1: LDL CH_3_–(CH_2_)n–
L2: VLDL CH_3_–(CH_2_)n–
L3: LDL CH_3_–(CH_2_)n–/VLDL CH_3_–(CH_2_)n–
L4: VLDL –CH_2_–CH_2_–C=O–
L5: CH_2_–CH=CH–
L6: Unsaturated lipids –CH=CH–
NAC1: *N*-Acetylated glycoprotein 1
NAC2: *N*-Acetylated glycoprotein 2
