# Supplementary figures and images for: Fusion of the 1H NMR data of serum, urine and exhaled breath condensate in order to discriminate chronic obstructive pulmonary disease and obstructive sleep apnea syndrome
Source: Metabolomics. 2015 May 22;11(6):1563–74. doi: 10.1007/s11306-015-0808-5 (PMC4605976; doi:10.1007/s11306-015-0808-5)

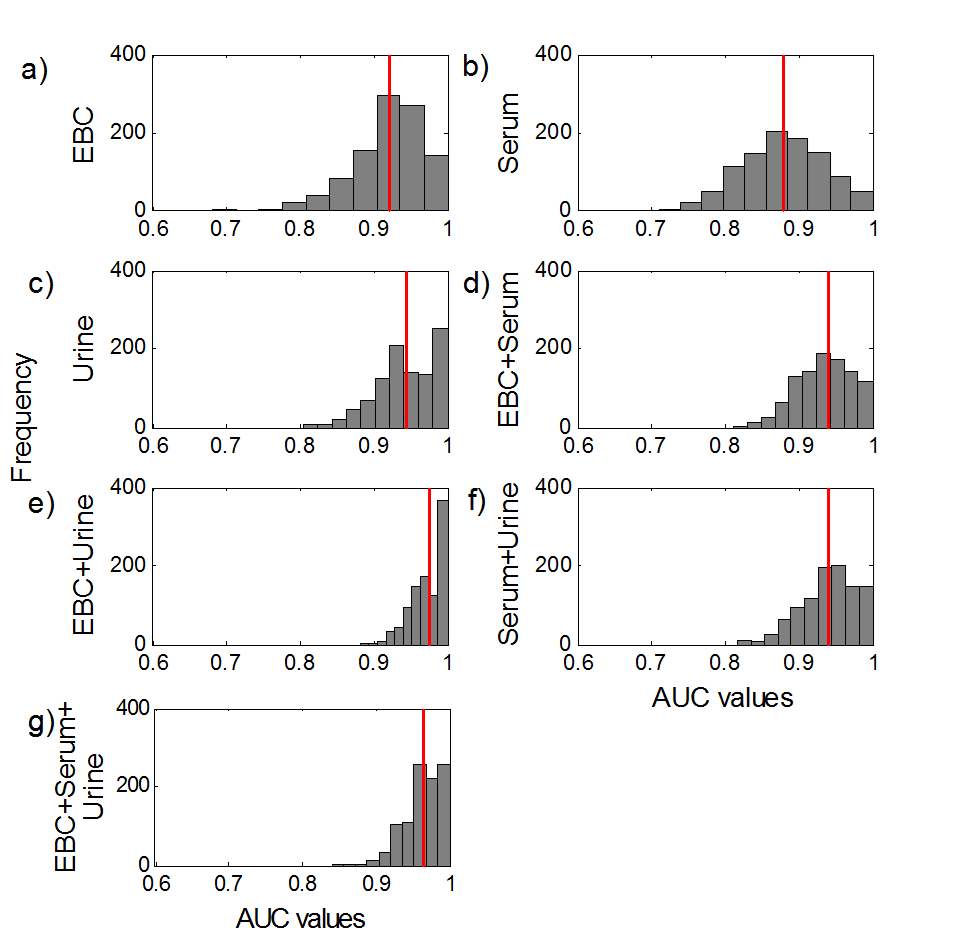

Supplement: Supplementary file 1 — Histograms of the AUC values that were obtained from the bootstrapped PLS-DA procedure using a) all EBC variables, b) all serum variables and c) all urine variables, d) EBC and serum variables, e) EBC and urine variables f) serum and urine variables and g) all three-block variables. The average AUC value is represented by the vertical red line. Supplementary material 1 (TIFF 176 kb) [file 11306_2015_808_MOESM1_ESM.tif]

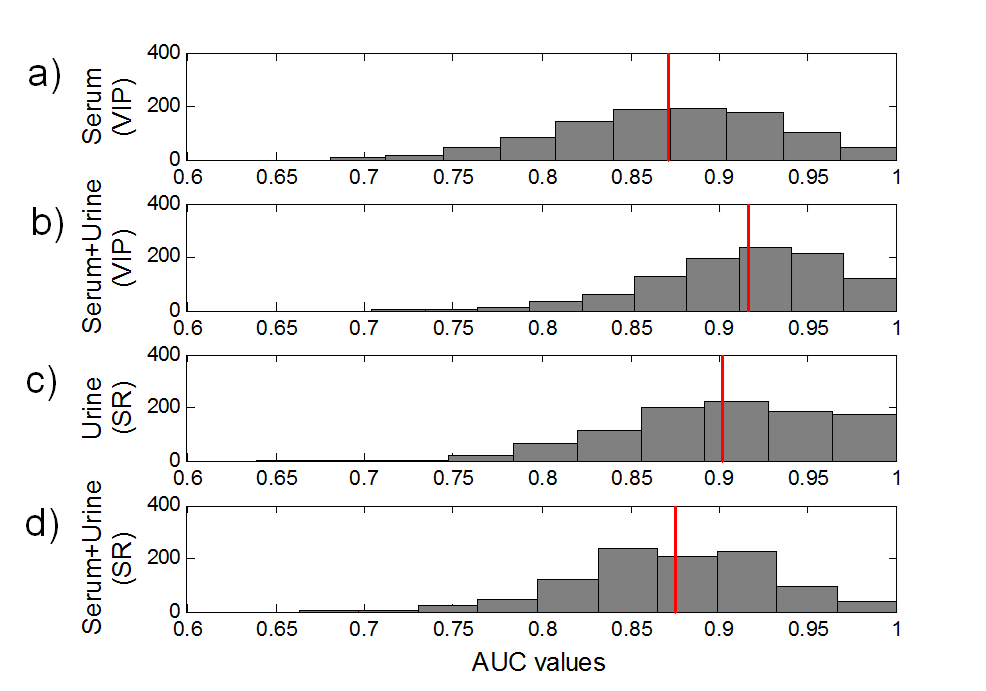

Supplement: Supplementary file 2 — Histograms of the AUC values that were obtained from the models with selected variables using a) the VIP-PLS-DA method of serum variables, b) the VIP-PLS-DA method of serum and urine variables, c) the SR-PLS-DA of urine variables and d) the SR-PLS-DA of serum and urine variables. Supplementary material 2 (TIFF 116 kb) [file 11306_2015_808_MOESM2_ESM.tif]
